# The Future of the Voluntary System

**Published:** 1919-07-05

**Authors:** 


### The Hospital

The Workers' Newspaper of Administrative Medicine and Institutional Life, Administration, National Insurance and Health.

No. 1726, Vol. LXVI. SATURDAY, JULY 5, 1919. PRICE TWOPENCE

### THE FUTURE OF THE VOLUNTARY SYSTEM.

There is a great deal of loose talk here and there about the future of the voluntary system of hospitals in this country, where freedom has continued so unchecked for centuries that to-day it militates against liberty, and is apt to degenerate into licence. It is certain that if any Government attempted to supplant the voluntary hospitals by establishing a system of State hospitals, with the official and disciplinary routine which must dominate such institutions, the people of all classes would soon rise in rebellion, for they would never consent to become mere material for science. Further, they cannot afford to relinquish the tender, loving care and personal service in hospitals which is guaranteed to the English people of all classes under the voluntary system. It is well, and has indeed become necessary, however, to raise a note of caution, and to stimulate to prompt action the responsible managers of every voluntary hospital throughout the United Kingdom.

On and from January 15, 1913, when the National Health Insurance Acts came into operation, it was estimated that fifteen million people, or thereabouts, would become entitled, as insured persons, to medical benefits under the Acts. It was estimated too by probably the ablest and most knowledgeable of all the insurance managers that of this number at least 600,000 per week, or four per cent., would at once require medical benefit. Further that the proportion of those requiring such benefit week by week would tend to increase in numbers, and not to diminish. Of this weekly minimum of 600,000 persons a large proportion would need and must somehow obtain hospital treatment. Mr. Lloyd George, although the matter was brought urgently to his notice, and specially represented to the Cabinet at the time, decided not to provide for the hospital requirements of insured persons, but to ignore them altogether. In the following year the Great War began, and this, with other important matters affecting insurance, has had to slide. In the result thousands of insured persons needing adequate treatment found to their disgust and loss that, instead of being able readily to obtain through National Health Insurance with assured certainty the medical aid of which they stood in need, had to seek it at their own cost from medical practitioners in whom they were justified, by experience, in placing full confidence. This position was aggravated by the absence of provision whereby insured persons could obtain definite hospital treatment, for this under the Act was non-existent and unprovided. Yet it was made perfectly clear to the Government of the day, and insisted upon in these columns, that the voluntary hospitals could not, without grave abuse, grant free medical relief to insured persons. British men and women were thus placed in a humiliating position, which Germans of both sexes had refused to occupy, that of being recipients of charitable relief, if and when they could obtain it, which in practice was seldom or never open to insured persons of both sexes resident in the United Kingdom. The problem of the degradation of the insured still remains unsolved and unsettled.

The amendments to the Insurance Acts since January 1913 have in no way provided adequately against the threatened abuses by insured persons of the voluntary hospitals, nor do they protect this class of insured person from the degradation before mentioned. There also remain the medical and business aspects to be considered. Up and down the country members of the medical staff of the voluntary hospitals have intimated that they are not prepared to give free medical relief indiscriminately, if at all, to any in or out-patient at a voluntary hospital, being an insured person. Portsmouth afforded a striking example, for there the leading members of the medical profession refused to grant free medical relief to insured persons who presented themselves as patients for free treatment at the voluntary hospitals. To-day, with increasing insistence, the difficulties in regard to hospital care still exist, and call loudly for an adequate remedy. With the signing of Peace the many momentous questions and difficulties created by the introduction of a great system of National Insurance into this country, which still remain to be considered and redressed, have made it imperative that not only the managers of the voluntary hospitals, but every thinking man and woman throughout the United Kingdom, should promptly consider the relative merits of State and voluntary hospitals. The more they do this, the sooner must they possess the knowledge that the voluntary system at its best is undoubtedly the most humane, least extravagant, and the most efficient hospital system the world has ever seen. It is the wonder and pride of our Allies and many nations, and the British people, in exact proportion to their intelligence, independence, knowledge, and self-respect, have become increasingly aware of late, that their best interests, and those of their children, require the speedy combination of many classes of the community to ensure for themselves and their families, the perpetuation in this free country, after the war, of the safeguards and privileges which they and their children have enjoyed under the voluntary system of hospitals.

The voluntary is the only system which absolutely guarantees, as experience demonstrates, that the patients' interest invariably become the first consideration, and that their need affords to each individual fortunate enough to obtain admission to a well administered voluntary hospital, the best, most efficient and loving care, a high standard of medical and surgical treatment, and the constant attendance of fully trained nurses of the best. All these considerations it is our privilege and duty to bring to the notice, at this time, of the inhabitants of Great Britain, that they may as a free people make the re-settlement of the hospital question, with its vital interests and importance, the greatest and most pressing task for all classes on the termination of the greatest war of the world, and the conclusion of peace.

